# An Environmentally Friendly Approach for the Fabrication of Conductive Superhydrophobic Coatings with Sandwich-Like Structures

**DOI:** 10.3390/polym10040378

**Published:** 2018-03-30

**Authors:** Xiaomin Luo, Wenjie Hu, Min Cao, Huijun Ren, Jianyan Feng, Mengyuan Wei

**Affiliations:** College of Bioresources Chemical and Materials Engineering, Shaanxi University of Science and Technology, Xi’an 710021, China; 18700821859@163.com (W.H.); caomincaomin1216@126.com (M.C.); renhj@sust.edu.cn (H.R.); fengjianyan@sust.edu.cn (J.F.); 8706770977@163.com (M.W.)

**Keywords:** environmentally friendly, conductivity, polyurethane

## Abstract

A large amount of research has been devoted to developing novel superhydrophobic coatings. However, it is still a great challenge to pursuean environmentally friendly method that leads to superhydrophobic coatings. Herein, we demonstrate for the first time, an environmentally friendly method for the preparation of conductive superhydrophobic coatings with sandwich-like structures by using aminoethylaminopropyl polydimethylsiloxane modified waterborne polyurethane (SiWPU) and *N*-octadecylamine functionalized multi-wall carbon nanotubes. These environmentally friendly coatings with the sheet resistance of 1.1 ± 0.1 kΩ/sq exhibit a high apparent contact angle of 158.1° ± 2° and a low sliding angle below 1°. The influence of the surface texture before and after heat treatment on the wetting properties is discussed. In addition, the coatings can be electrically heated by 3~113 °C with a voltage of 12~72 V, and thus, can be used for deicing. Furthermore, the resulting coatings demonstrate good performance of wear resistance and ultraviolet resistance, which will have broad application potential in harsh environments.

## 1. Introduction

Recently, surfaces with superhydrophobic properties have attracted extensive interest due to their potential applications, including self-cleaning, anti-icing, anti-bacteria, and anti-adhesion [1,2,3,4,5,6]. A variety of techniques, such as the etching method, electrospinning method, chemical vapor deposition method, self-assembly method, sol-gel method, electrodeposition method, hydrothermal method, and phase separation method, have been developed to fabricate superhydrophobic surfaces with different structures [7,8,9,10,11,12,13]. However, during the preparation process of these superhydrophobic coatings or surfaces, harmful organic solvents (such as chloroform, tetrahydrofuran, dimethyl formamide, acetone, and toluene) were used to disperse the nanoparticles or dissolve the organic components [14,15,16]. In addition, in some superhydrophobic surfaces, fluoride-rich materials have been adopted to enhance the hydrophobic effects [17,18]. These less eco-friendly materials not only contaminate the environment and do harm to the health of producers and users, but also increase the treatment cost of the pollutants and the production cost.

Although the theoretical study and preparation methods of superhydrophobic surfaces have made great progress, it is still a great challenge for the researchers to fabricate superhydrophobic surfaces without the use of organic components and fluoride-rich materials. So, it is necessary to develop a novel technology system to eliminate the pollutants fundamentally. Until now, the reported preparation methods of environmentally friendly superhydrophobic surfaces are mainly the electrochemical deposition method [19], the electrochemical etching method [20], femtosecond laser irradiation [21,22], the hot pressing method [23,24], and spraying polymer nanocomposites and waterborne suspensions [25]. Among these techniques, the spray method is attractive since it is fast, can be highly automatized on an industrial scale, and is without any restriction on substrate categories, which make it most likely to realize industrialization. It seems paradoxical that hydrophobic coatings are fabricated from waterborne polymer emulsion, because it was generally believed that hydrophobic polymer could not dissolve into water. But the fact is that researchers are able to use these hydrophobic polymers (e.g., fluorinated polyacrylate and organic silicon polyurethane) with carboxyl groups simultaneously. After ionization of the carboxyl groups, this kind of polymer is similar to polymeric surfactants, which can form in the micelle with the carboxyl arranged outward and the hydrophobic chain segment wrapped inward. This form of oil in water exists stably in the aqueous media. During drying, with the evaporation of water, the emulsion was gradually demulsified. Meanwhile, the micelle structure was damaged, and the hydrophobic chain segment was enriched toward the two ends of air so that a hydrophobic surface was ultimately generated. Therefore, this type of waterborne polymer emulsion provides a new way of fabricating environmentally friendly superhydrophobic coatings. Schutzius et al. [26] fabricated the superhydrophobic composite coatings on a variety of substrates by spraying water-based polyolefin-exfoliated graphite nanoplatelets dispersion. Aslanidou et al. [27] prepared the superhydrophobic protective coatings on silk by spraying water-soluble siloxane emulsion enriched with silica nanoparticles, without the use of any organic solvent. Similarly, Chatzigrigoriouet et al. [28] fabricated the superhydrophobic coatings by the same way. Raoet et al. [29] obtained waterborne self-healing superhydrophobic coatings by mixing fluoroalkyl silane (FAS)-loaded microcapsules, photocatalytic TiO_2_ nanoparticles, and FAS-modified SiO_2_ nanoparticles with waterborne polysiloxane resins. Chen et al. [30] also prepared all water-based self-repairing, superhydrophobic coatings by the same way. Mates et al. [25] demonstrated a water-based superhydrophobic coating on nonwoven and cellulosic substrates by spraying bentonite nanoclay and aqueous fluoroacrylic copolymer dispersions. Milionis et al. [31] presented a simple, one-step, water-based spray coating process to obtain superhydrophobic and superoleophobic coatings on metals comprising hydrophilic silica nanoparticles and fluoroacrylic polymer. Although these methods avoided the use of organic solvents, some of them still used fluoride-rich materials, which would bring about new contamination. Moreover, in these methods, nanoparticles and polymer waterborne dispersions were directly mixed together and thus led to poor dispersion of nanoparticles in polymer dispersions. As well, the addition of nanoparticles could lead to a sharp increase in viscosity of the composition system so as to easily block the spray head.

We report herein, for the first time, an environmentally friendly approach to fabricate conductive superhydrophobic coatings with sandwich-like structures. Firstly, aminoethylaminopropyl polydimethylsiloxane modified waterborne polyurethane (SiWPU) dispersion was successively sprayed onto glass slides and thoroughly dried. Then, ethanol dispersion of multiwalled carbon nanotubes (MWCNTs) was successively sprayed onto the SiWPU coatings and dried out completely. Next, SiWPU dispersion was sprayed onto the MWCNTs coatings and thoroughly dried. Finally, through heat treatment of the composite coatings, superhydrophobic coatings were obtained. So far, it has not been reported that waterborne polyurethane modified with silicone was used to fabricate superhydrophobic coatings.

To improve the dispersibility and stability of the MWCNTs in ethanol, *N*-octadecylamine with a long aliphatic chain was firmly grafted to the surface of the MWCNTs. In the process of the fabrication of the superhydrophobic coatings, only ethanol and water were used as dispersion media, and all materials used were fluoride-free. So, this novel technology eliminated the pollution from headstream. Compared with the polymer/MWCNTs composite coatings fabricated by physical mixing method, in this system, the MWCNTs were dispersed evenly, and thus, the coating showed great conductivity and good electric heating characteristics. In addition, this conductive superhydrophobic coating is wear resistant and ultraviolet (UV)-resistant, so it has good prospects of application.

## 2. Materials and Methods

### 2.1. Materials

Polyoxytertramethylene (PTMG, *M*n = 2000) and isophorone diisocyanate (IPDI) were purchased from Aladdin Regent Co., Ltd., (Shanghai, China). Dimethylol propionic acid (DMPA) was purchased from Huada Industry Co., Ltd., (Yantai, China). Triethylamine (TEA) was obtained from Ginna Shijiazhuang Chemical Co., Ltd., (Shijiazhuang, China). Aminoethylaminopropyl polydimethylsiloxane (AEAPS) was purchased from Batai Chemical Co., Ltd., (Guangzhou, China). MWCNTs (>90% carbon basis) were obtained from Chengdu Organic Chemical Co., Ltd., (Chengdu, China) of the Chinese Academy of Sciences. The length of the MWCNTs was 10~30 µm with a diameter of 20~40 nm. *N*-octadecylamine (ODA) with a purity of 95% was purchased from Aladdin Regent Co., Ltd.

### 2.2. Preparation of the SiWPU (WPU-AEAPS) Emulsion

The synthesis of hydrophobic waterborne polyurethane (WPU) was a key part of our strategy. IPDI (8.34 g), PTMG (30.0 g), and dibutyltin dilaurate (0.5 g) were added into a four-necked flask equipped with a thermometer and an electric stirrer in a nitrogen atmosphere, reacting at 85 °C for 1.5 h. DMPA (1.15 g) was added subsequently, and the reaction was continued at 90 °C for 2 h. After the temperature was cooled to 55 °C, TEA (0.86 g) was added and stirred for 0.5 h. Next, AEAPS (2.4 g) was added dropwise into a flask and stirred for 1 h to modify the polyurethane (PU) prepolymer. Finally, the modified prepolymer was added into deionized water in a high-speed disperser (2000 rpm) and then was stirred rapidly for 1 h. Thus, the WPU-AEAPS emulsion was obtained.

### 2.3. Preparation of ODA-Functionalized MWCNTs

**Scheme 1:**
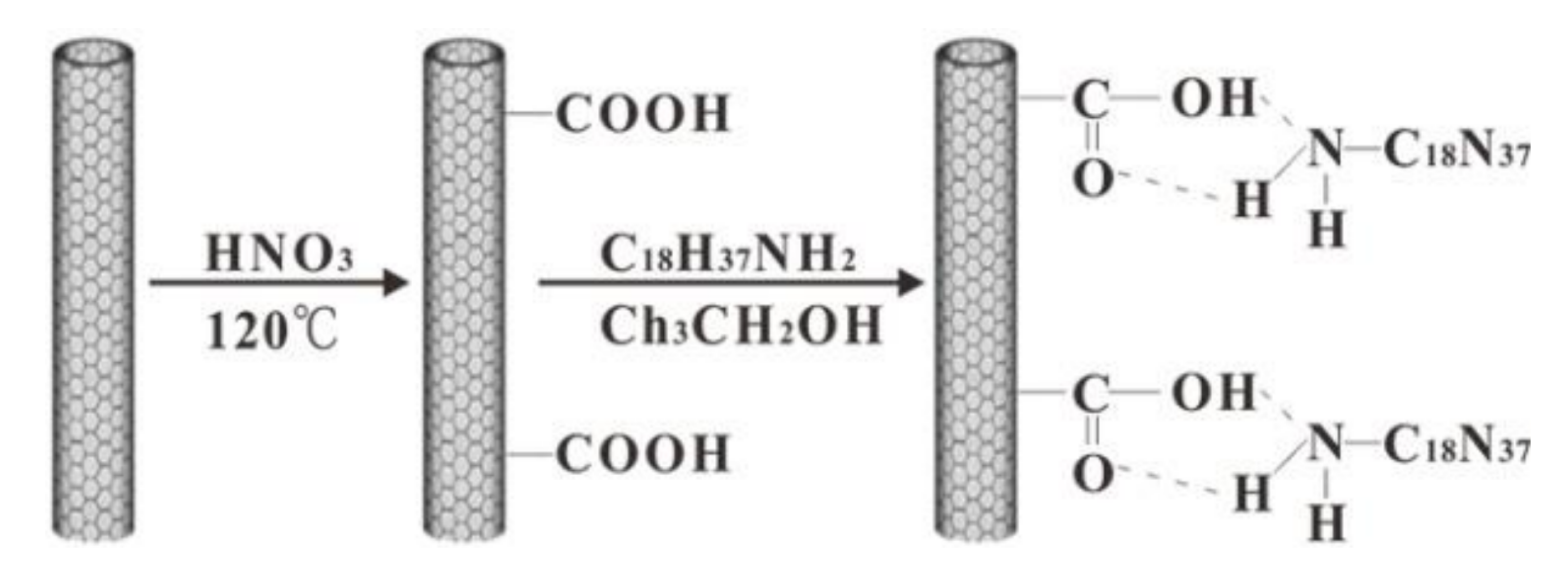
Grafting of *N*-octadecylamine to the multiwalled carbon nanotubes (MWCNTs).

A 500 mL flask charged with 6.0 g crude MWCNTs and 300 mL of HNO_3_ aqueous solution was sonicated in a bath (40 kHz) for 30 min. The mixture was then stirred for 24 h under reflux. After cooling to room temperature, it was diluted with 700 mL of deionized water and then was vacuum-filtered through a 0.22 μm cellulose membrane. The solid was washed with deionized water until the pH of the filtrate reached 7. The filtered solid was then dried under vacuum at 60 °C for 24 h to get 4.35 g (~70%) of carboxylic acid-functionalized MWCNTs (MWCNTs-COOH). Then, 1.0 g MWCNTs-COOH and 2.0 g *N*-octadecylamine were added into 60 mL of ethanol and were sonicated for 1 h to obtain a well-dispersed suspension. Then the mixed dispersion was refluxed under 78 °C for 24 h. The resulting product was centrifuged at 9000 rpm for 5 min and washed thoroughly with ethanol to remove the remaining ODA. After drying at 60 °C for 12 h, ODA-functionalized MWCNTs (MWCNTs-ODA) were obtained (shown in Scheme 1).

### 2.4. Preparation of Superhydrophobic Coating with Sandwich-Like Structures

The SiWPU emulsion described in Section 2.2 was sprayed onto a glass slide using an air gun. The spraying distance was approximately 20 cm with a 2-bar gauge air pressure. After spraying the coating, the sample was left to dry at 60 °C for 30 min. Then, the MWCNTs-ODA ethanol dispersion with acontent of 1% was sprayed onto the previously coated SiWPU layer and the ethanol evaporated under ambient conditions for 10 min. This process was repeated five times. Then, the SiWPU emulsion was again sprayed on the coated MWCNTs-ODA layer, and the composite with sandwich-like structures of “SiWPU-MWCNTs-SiWPU” was obtained. Upon thermal treatment of the nanocomposites slightly above the viscous flow temperature of the WPU polymer (about 150~160 °C), the composite coatings became superhydrophobic.

### 2.5. Characterizations

The infrared spectroscopy was characterized by an American Brooke’s Verte-70V Fourier Transform Infrared Spectrometer (Brooke, Madison, WI, USA). Thermogravimetric Analysis was characterized byan American TA Instruments Q500 (TA Instruments, New Castle, DE, USA), after the sample was dried in vacuo. X-ray photoelectron spectroscopy (XPS) measurements were carried out on a Thermo Scientific K-Alpha XPS spectrometer (Thermo Fisher Scientific, West Palm Beach, FL, USA). The morphology of the coatings was tested using a field emission scanning electron microscope (FESEM) (Hitachi, FE-SEM S4800, Tokyo, Japan). Contact angle (CA) and sliding angle (SA) of the superhydrophobic coating were measured using a 10 μL deionized water droplet on a video optical contact angle system (OCA 20, Dataphysics, Filderstadt, Germany) at room temperature. The CA and SA were measured on five different sites of each sample. The mean value was taken as the final result. The conductivity of the resulting coating was measured using a standard four-probe sheet resistance measurement (SZT-2B, Suzhou, Tongchuang, China). Thermal infrared images were recorded by a FLUKE TiS10 thermal infrared camera (Brooke, Madison, WI, USA). The wear resistance testing method is listed below. The coatings weighing 100 g were placed face-down on sandpaper with 600 meshes and pushed forward 10 cm, which was a cycle. After each complete cycle, the contact angle was measured.

## 3. Result and Discussion

**Figure 1:**
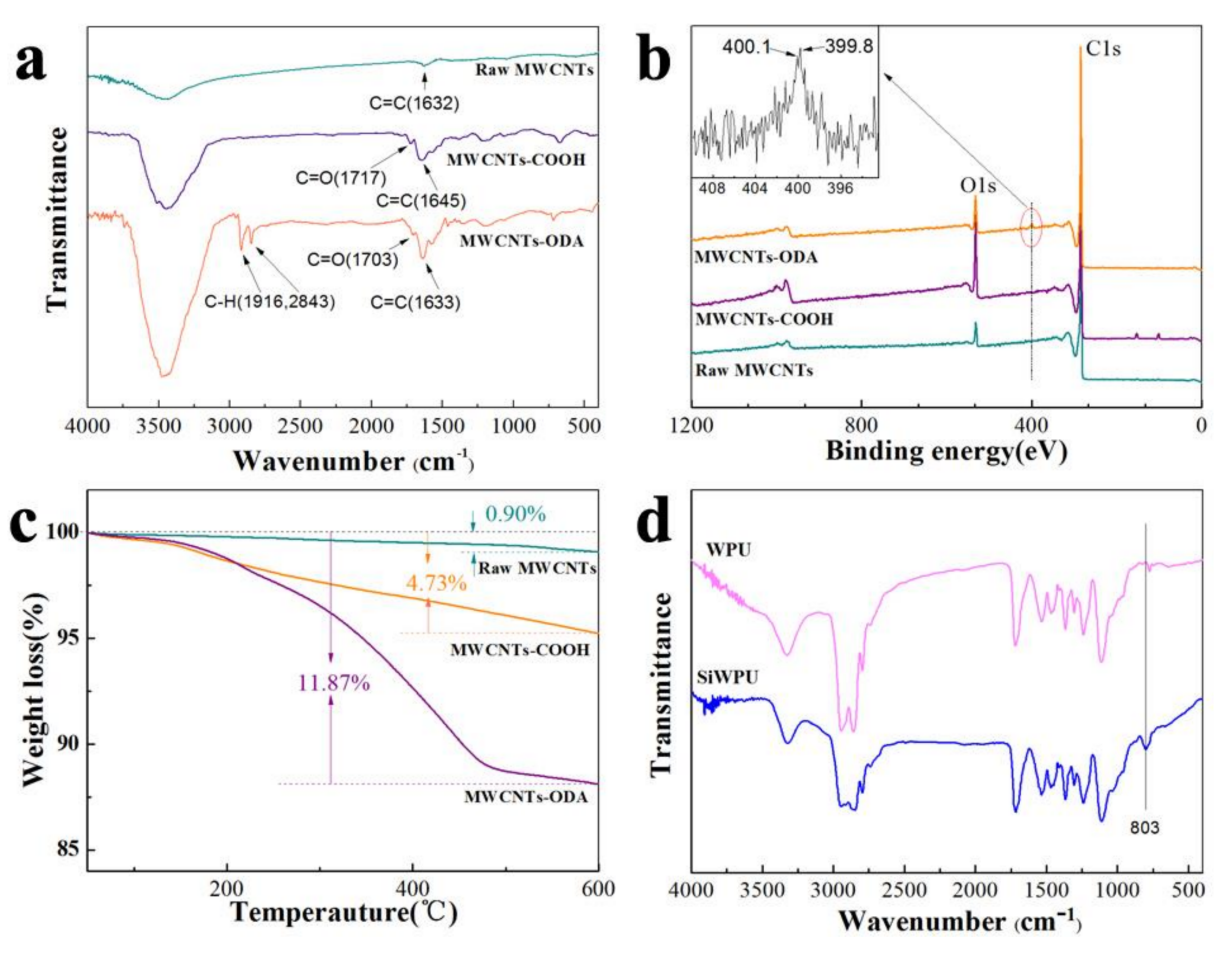
FTIR spectra (**a**), X-ray photoelectron spectroscopy (XPS) survey spectra (**b**) and TGA curves (**c**) of raw MWCNTs, carboxylic acid-functionalized MWCNTs (MWCNTs-COOH), and ODA-functionalized MWCNTs (MWCNTs-ODA). FTIR spectra (**d**) of waterborne polyurethane (WPU) and aminoethylaminopropyl polydimethylsiloxane modified waterborne polyurethane (SiWPU).

Herein, conductive superhydrophobic coatings were fabricated by using *N*-octadecylamine grafted onto the MWCNTs and AEAPS-modified waterborne polyurethane. To improve the stability and dispersion of the raw MWCNTs in ethanol, MWCNTs-ODA was fabricated. As shown in Scheme 1, *N*-octadecylamine grafted onto the MWNCTs was firstly obtained via a similar process. FTIR and XPS were used to monitor the process (Figure 1a,b).

As can be seen in the infrared spectra, the raw MWCNTs show peaks with quite low intensity at 3466 cm^−1^, which can be ascribed to the O–H vibration, because the MWCNTs hold water and oxygen easily in air [32]. For the spectrum of the MWCNTs-COOH, the band at 3435 cm^−1^ is strengthened and a new band appears at 1717 cm^−1^, assigning to the C=O stretching vibration, indicating that a carboxyl group has been introduced on the surface of the MWCNTs after the nitric acid treatment [33]. In the spectrum of the MWCNTs-ODA, the prominent peaks at 2916 and 2843 cm^−1^ correspond to the CH_3_– and –CH_2_– bond vibrations in the *N*-octadecylamine, which shows that the *N*-octadecylamine is successfully grafted onto the MWCNTs. Figure 1a also shows that the prominent C=O peak of the MWCNTs-ODA red shifts from 1717 to 1703 cm^−1^, which is attributed to the formation of a hydrogen bond between the C=O and N–H groups [34]. Meanwhile, the fact that O intensity at 532.6 eV of the MWCNTs-COOH is much higher than that of the raw MWCNTs, which indicates that the raw MWCNTs have been oxidized by the concentrated HNO_3_, and the appearance of obvious N signals at 400.1 eV with a lower-binding-energy shoulder at 399.8 eV indicates that the *N*-octadecylamine grafted onto the MWCNTs have been successfully obtained according to the process shown in Scheme 1.

TGA measurement has provided further evidence for the grafting of the *N*-octadecylamine to the MWCNTs. Figure 1c shows their TGA curves. The raw MWCNTs have only a 0.9% weight loss in the range between 50 and 600 °C. However, the MWCNTs-COOH has a 4.73% weight loss. The 3.83 wt % weight loss can be assigned to the decomposition of the grafted carboxyl groups. For the TGA curve of the MWCNTs-ODA, there is a 11.87% weight loss in the range of 50–600 °C, and a 7.14% weight loss can be used to appropriately estimate the mass percentage of the *N*-octadecylamine grafted onto the MWCNTs [32].

In Figure 1d, for WPU and SiWPU, N–H stretching vibration peaks in the ureido are at 3324 cm^−1^, C=O stretching vibration peaks in the ureido are at 1716 cm^−1^, and N–H deformation vibration peaks in the ureido are at 1530 cm^−1^. The appearance of the above three absorption peaks indicates that there exist carbamic acid ester groups. At 2270 cm^−1^, both do not show –CNO’s characteristic absorption peaks, indicating that –CNO is completely reacted. Around 803 cm^−1^, SiWPU shows that the characteristic absorption peak of CH_3_ symmetrical deformation vibration belongs to Si–CH_3_, indicating that AEAPS has successfully grafted onto the WPU chain segment [35].

**Figure 2:**
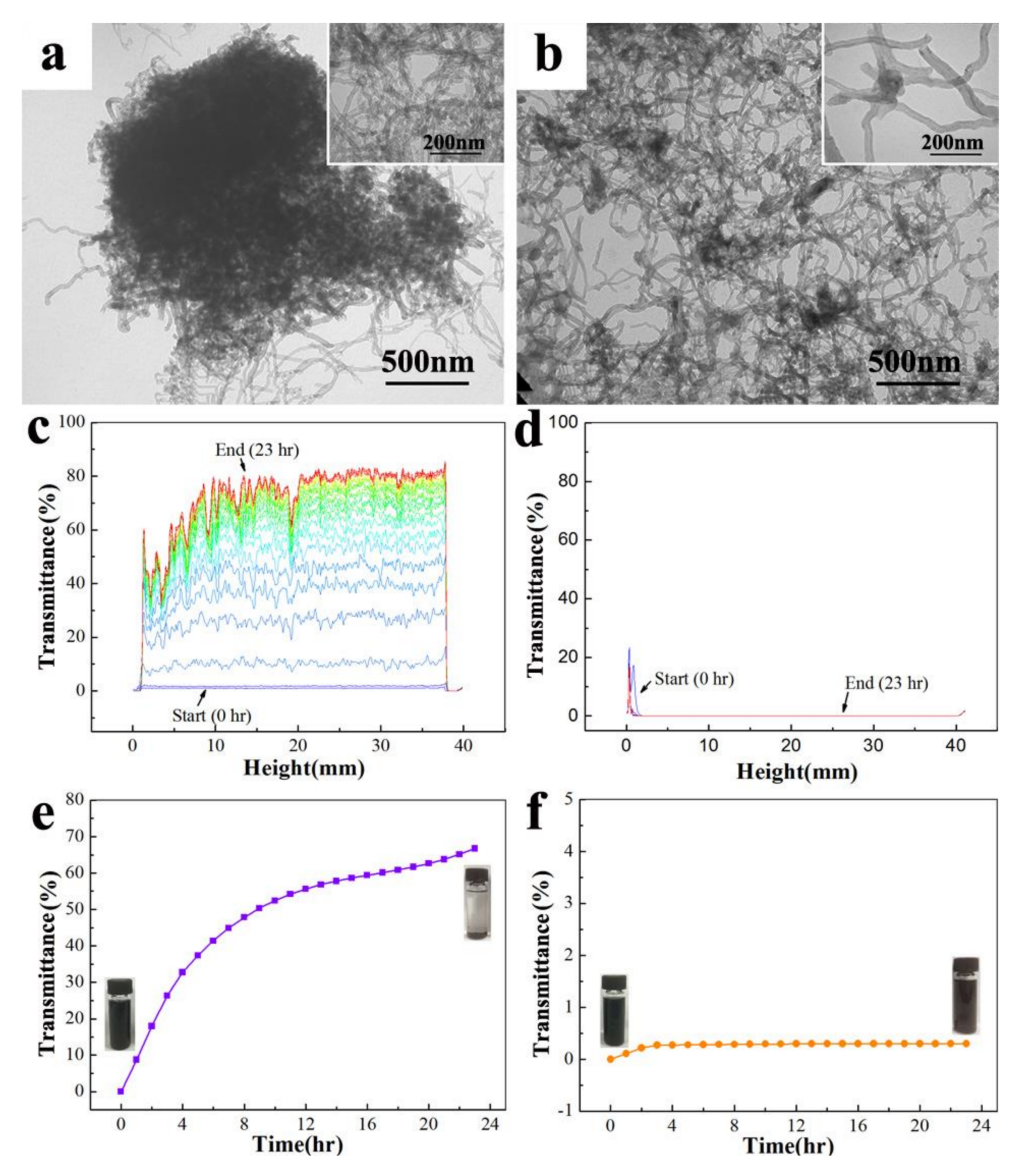
TEM images, turbiscan data and kinetic motion of fluctuations by means of time of the raw MWCNTs and the MWCNTs-ODA in ethanol, (**a**,**c**,**e**) raw MWCNTs, (**b**,**d**,**f**) MWCNTs-ODA. The insets (**a**,**b**) are the magnified images of the raw MWCNTs and the MWCNTs-ODA, respectively.

Usually, the dispersibility and the stability of nanoparticles in an organic solvent determine whether to generate the uniform and stable nano dispersion or not, and even determine whether to form the homogeneous and well-dispersed nano coating on the surface of the substrate. In the present study, we explored the dispersibility and the stability of the MWCNTs in ethanol before and after modification by TEM and Turbiscan Lab, respectively. From Figure 2a, it shows that opaque agglomerates formed by the raw MWCNTs appear. In its high-resolution images, a single MWCNT even cannot be observed. The appearance of agglomerates is mainly due to the quantum-size effect of the MWCNTs. Moreover, as one-dimensional materials, MWCNTs have a large aspect ratio, and thus, the numerous MWCNTs are easily mingled and tangled together. So, it is quite difficult for these agglomerates to disperse because the two effects integrated with each other. However, after being modified by *N*-octadecylamine, the dispersibility of the MWCNTs in ethanol has been improved obviously, and the tube diameter of the MWCNTs becomes clearly bigger (the inset of Figure 2b). This is because long alkyl chains are formed on the surface of the MWCNTs grafted by octdecylamine, which improves their solubility in ethanol so as to improve their dispersibility.

The stability of the MWCNTs before and after modification can be tested by a universal stability analyzer. The mass fraction of test dispersions of the raw MWCNTs and the MWCNTs-ODA in ethanol is 0.02%, and the two samples are respectively sonicated for 1 min before testing. It is set to scan once 1 h, and there are a total 24 times of scanning. The stability of the MWCNTs in ethanol is measured with the transmittance of a near-infrared light pulse (λ = 800 nm). Figure 2c,d show the transmittance curves of the MWCNTs and the modified MWCNTs in ethanol dispersions at different times, respectively. It is known from the results that the settling rate of the raw MWCNTs in ethanol is very fast, and the transmittance of the dispersion is increased by over 66.7% after 23 h, while the settling rate of the MWCNTs-ODA is very slow and the transmittance is increased by only 0.2% after 23 h. Figure 2e,f show the mean transmittance of the raw MWCNTs and MWCNTs-ODA every hour. Figure 2 shows that within 4 h the settling rate of the raw MWCNTs is quite fast, but after 10 h, the transmittance of the dispersion tends to be stable. Whereas the settling rate of the MWCNTs-ODA is faster within 2 h, after 3 h, the transmittance of the dispersion tends to be stable. This is due to the lack of active functional groups on the surface of the raw MWCNTs, which thus leads to poor solubility in all kinds of solvents. After being modified by *N*-octadecylamine, long chain alkyl groups are generated on the surface of the raw MWCNTs, which greatly improve their solubility in ethanol so as to form stable dispersion.

**Figure 3:**
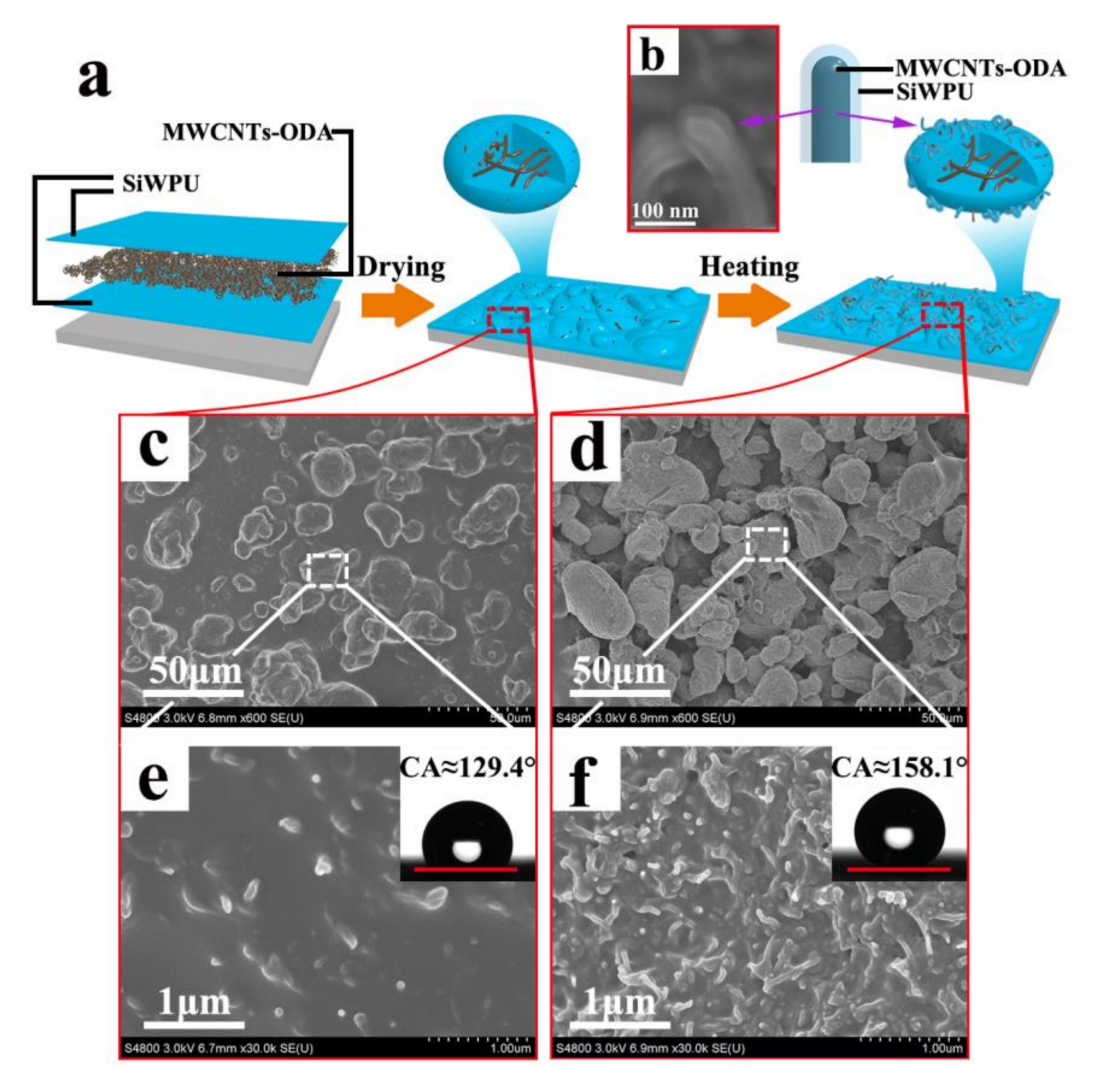
(**a**) Schematic diagram of the fabrication of conductive superhydrophobic coatings with sandwich-like structures. (**b**) scanning electron microscope (SEM) surface images of nanocomposite coatings before and after thermal treatment. The inset (**b**) is the profile of water on the nanocomposite coating. (**c**,**e**) SEM images of nanocomposite coatings before thermal treatment. (**d**,**f**) SEM images of nanocomposite coatings after thermal treatment.

The conductive superhydrophobic coating with sandwich-like structures of “SiWPU-MWCNTs-SiWPU” is fabricated via the spraying method and further heat treatment, as is shown in Figure 3a. From scanning electron microscope (SEM) images, it can be known that a large number of irregular micrometer-scale papillae are formed on the surface of the composite coatings sprayed by the SiWPU emulsion, the MWCNTs ethanol dispersion, and the SiWPU emulsion successively. The sizes of these papillae are between 13.1 and 35.7 μm (Figure 3c). The formation of them is due to the rapid volatilization of water in the drying process. When the SiWPU emulsion is sprayed on the MWCNTs coating, both are mixed physically to form a viscous SiWPU/MWCNTs mixture. During drying, with the volatilization of water, the concentrations of SiWPU and MWCNTs in the droplets are increased rapidly and their viscosity is sharply increased. When these poor fluidity droplets contact the polyurethane coating at the bottom, they cannot spread out rapidly on the surface. In the meantime, the countless, highly viscous droplets are aggregated and adhered to one another. With the complete volatilization of water, the micrometer-scale papillae are gradually formed. In high-magnification images, it can be seen that a few MWCNTs are distributed on the surface (as shown in Figure 3e), while a majority of the MWCNTs are wrapped into the papillae. Although the micrometer-scale papillae are produced on the surface, the composite coating fails to reach superhydrophobicity due to the small roughness of surface. The contact angle of these surfaces is 129.4°, and the droplets are still fastened to the surface, which shows the Wenzel state.

Jiang Lei et al. [36] found that there are nanoscale hierarchical structures in the micrometer-scale papillae on the lotus leaf surface by observing the microstructure of the lotus leaf surface. The micro- and nanoscale hierarchical structure is responsible for the superhydrophobicity of the lotus leaf surface. From the observation of the lotus leaf microstructure, it can be learned that to fabricate the surface with superhydrophobicity, these micrometer-scale papillae also need to have nanoscale hierarchical structures on them. In order to cause the MWCNTs to bulge out within the micrometer-scale papillae, the composite coating is heated over the viscous flow temperature point of the polyurethane (about 150~160 °C) and kept at a certain time to make polyurethane resin fuse and flow toward the interior of the skeleton of the MWCNTs so that a nanoscale structure tangled with the MWCNTs is formed on the micrometer-scale papillae (Figure 3f). Moreover, it can be observed from SEM (as shown in Figure 3b) that while the polyurethane resin is fused to flow downward, it can wrap the MWCNTs partially. After heat treatment, the microstructure of the composite coating surface resembles the lotus leaf surface, which is covered with SiWPU and octdecylamine with low surface energy, showing the superhydrophobicity. The water contact angle reaches 158.1° ± 2°, and the sliding angle is below 1°.

**Figure 4:**
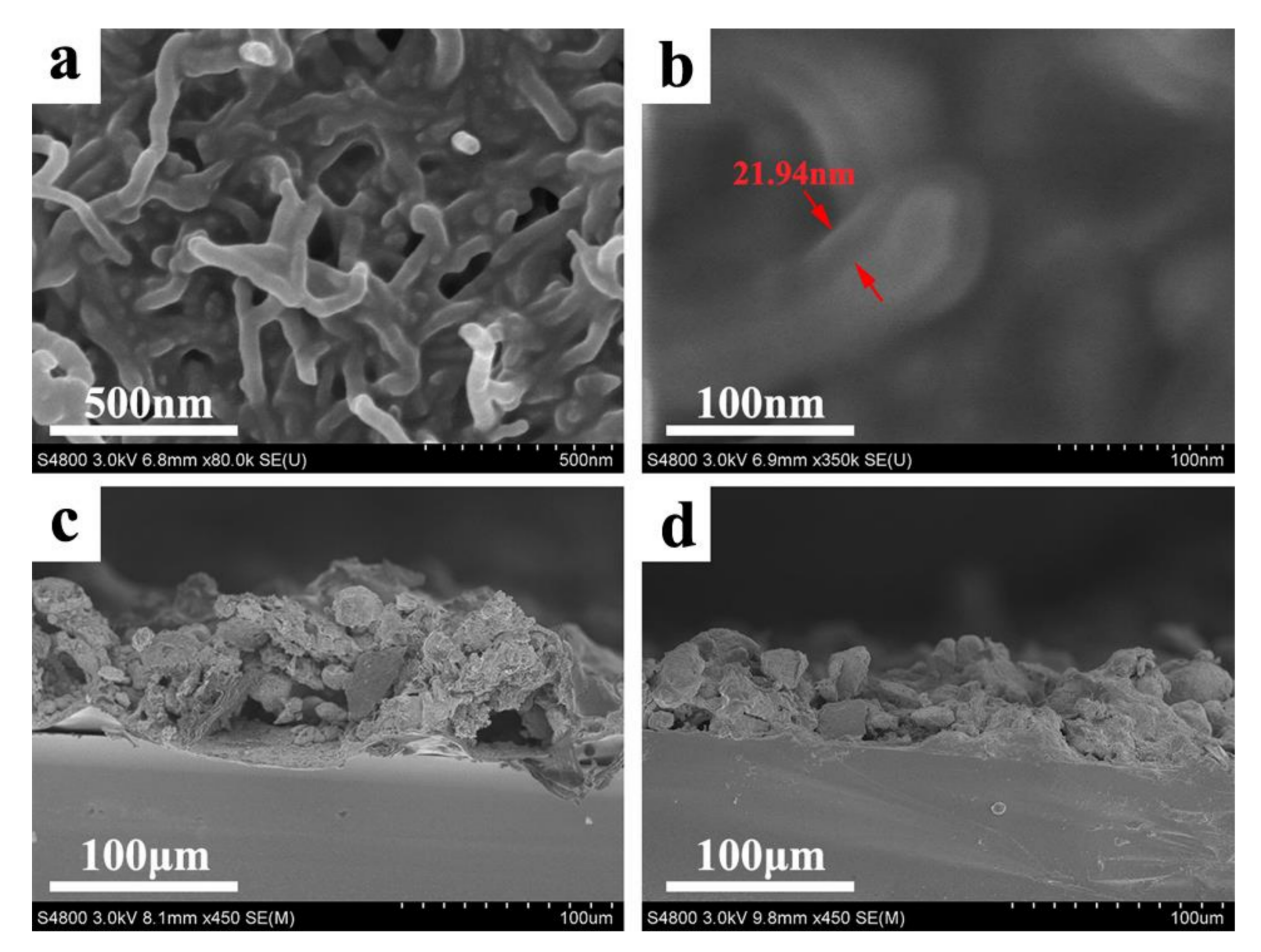
(**a**,**b**) SEM images of the nanocomposite coatings after thermal treatment at different magnifications. Morphology of the cross-sectional of the nanocomposite coatings, (**c**) before thermal treatment, and (**d**) after thermal treatment.

Based on the Cassie equation [37], if the solid surface has only one rough structure, the apparent contact angle (θ*) only depends on the solid phase fraction. From Figure 3d, it is known that after heat treatment, a large number of micron-scale cavities are generated between the micron papillae on the surface of the composite coating. And nano-scale cavities are formed on every papilla (Figure 4a). In these micron-/nano-scale cavities, a lot of air is embedded, and thus, the composite coating has extremely low solid fraction. Meanwhile, the rough surface covered with SiWPU and ODA has low surface energy so that the composite coating shows the superhydrophobic property. In addition, it is also observed that the MWCNTs is wrapped by SiWPU, and the wrapping thickness is about 20 nm. Figure 4c,d are the sectional SEM images of the composite coating before and after heat treatment. From Figure 4c, it is known that the coating thickness before heat treatment is about 50~70 μm, and the polyurethane coating at the bottom can be clearly observed. Because the superficial polyurethane coating is mixed with the MWCNTs physically, the clear interface layer cannot be seen. After heat treatment, because the superficial polyurethane layer flows toward the interior of the MWCNTs coating, the thickness of the whole becomes thinner, and the thickness is about 30~50 μm. In addition, it can also be seen that in the composite coating after heat treatment, the interface at the bottom of the polyurethane resin layer disappears. This is because during heating the polyurethane resin is fused, spread out again, and combined to the substrate closely. This fusion and recombination are favorable to increasing the binding force of the composite coating with the substrate.

**Figure 5:**
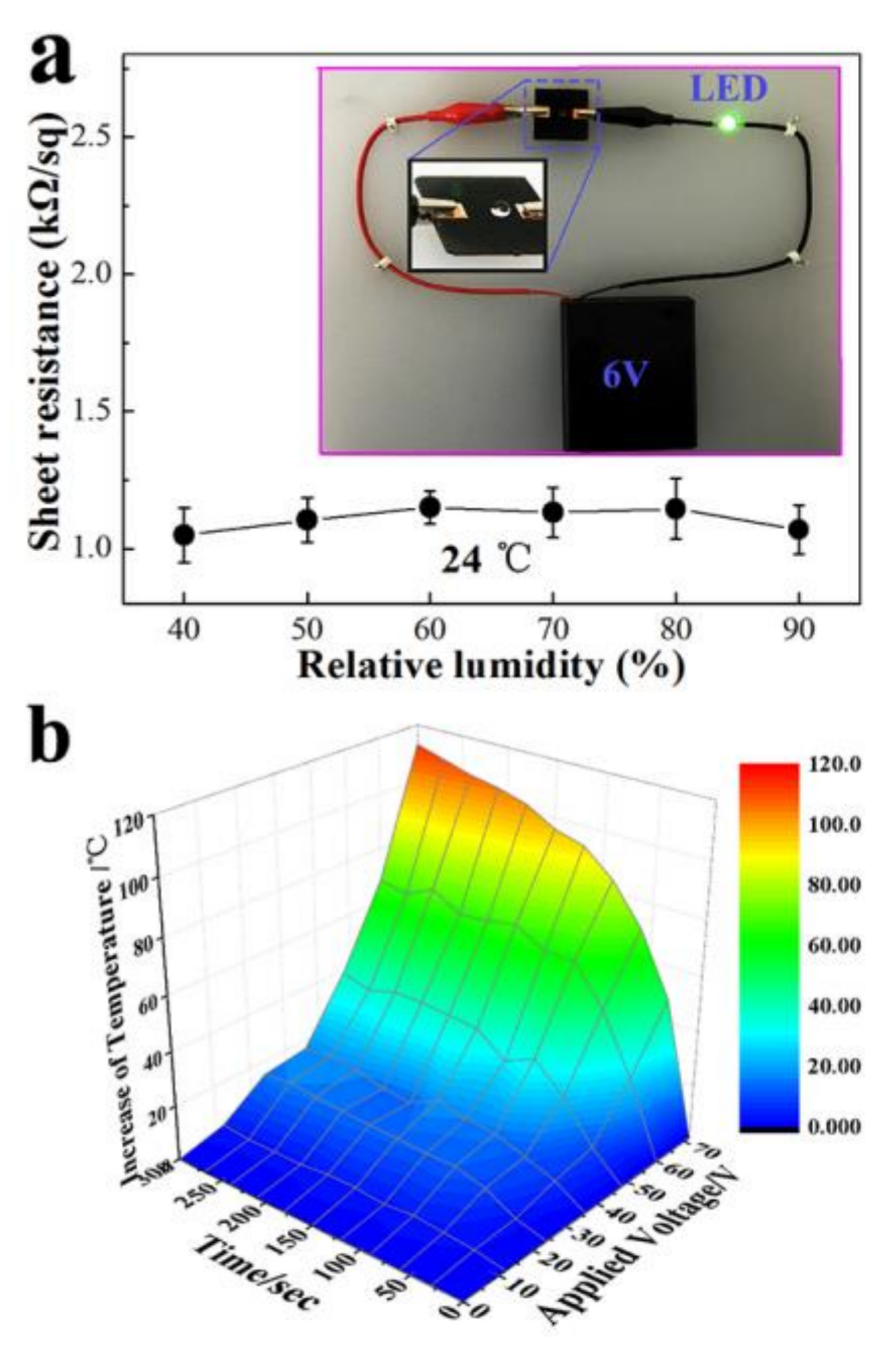
(**a**) Correlation between the resistance values of the superhydrophobic coatings and the relative humidity at 24 °C; (**b**) The increase of temperature at the center of the superhydrophobic coatings under different voltages.

It is generally known that a lot of air is embedded into the roughness structure of the superhydrophobic surface and generates an air layer. When exposed to supercooled water, the superhydrophobic surface will make the water droplets bounce so as to delay or even prevent the formation of snow, ice, and frost on the surface. However, once ice comes, the superhydrophobic surface usually loses its superhydrophobic properties due to the damage of the micro-nanometer hierarchical structure on the surface. Therefore, if we endow the superhydrophobic coatings with conductivity and utilize electric heating characteristics to remove ice, the above problems are readily solved. According to the literature [38], carbon nanotubes (CNTs) are an ideal conductive filler, and their recombination with polymer resins can obviously increase the conductivity of the composite material. The test results of the sheet resistance of superhydrophobic coatings under different humidities are shown in Figure 5a. It is known that with the increase of relative humidity from 50% to 90%, the sheet resistance values are changed within a fairly small range, about 1.1 ± 0.1 kΩ/sq, indicating stable conductivity. Furthermore, when the conductive superhydrophobic coatings are connected in series to the circuit, the light-emitting diode (LED) was lightened under the low voltage of only 6 V, as shown in Video S1. Usually, polymer-carbon nanotube composite materials fabricated by the physical mixing method possess poor conductivity, because of high viscosity results from the large, specific surface area of the MWCNTs, which reduce the conductivity of the composite material remarkably. Compared with the physical mixing method, the preparation method in this paper effectively avoids this problem by step-by-step spraying of the MWCNTs dispersion and resin emulsion. The connection between the MWCNTs is closer, and it is easier to form a 3D f conductive circuit so that the fabricated coatings exhibit excellent conductivity.

The electric heating characteristic of the conductive superhydrophobic coating has been studied by the following method. First, precisely two pure copper alligator clips are put on the two sites at a distance of 2 cm on the composite surface. Then, a wire is used to connect the two alligator clips, an adjustable transformer, and the power source in series to form a direct current circuit. Next, the adjustable transformer is used to exert the different applied voltages on the composite coating while the surface temperatures are detected by a thermal infrared camera at different times. Figure 5b shows the results of the temperature variation of the composite coating surface with the heating time at different applied voltages. As shown, within a certain period, the temperature of the composite coating is increased exponentially while the applied voltage is increased linearly. And under a certain applied voltage, as the heating time goes on, the temperature of the composite coating is increased first, and then tends to return to a certain value, which corresponds to Joule’s law *Q* = (*V*^2^/*R*)*t*. That is to say, for the constant sheet resistance, the quantity of heat is closely related to the voltages on the two ends and the heating time. When the applied voltage is increased to 72 V and kept for 5 min, the surface temperature of the composite coating will reach 113 °C, showing good electric heating characteristics. Moreover, the composite coating maintains its superhydrophobic property, no matter how variable the surface temperature. The contact angle falls slightly. When the surface temperature of the composite coating is over the boiling point of the water droplets, the droplets are rapidly evaporated, and the coatings are ultimately kept in the dry state. However, the applied voltage must be controlled below the viscous flow temperature point of the polyurethane resin, otherwise the coatings are damaged easily. These superhydrophobic coatings with low adhesion and electric heating characteristics have a promising future in the field of ice resistance.

**Figure 6:**
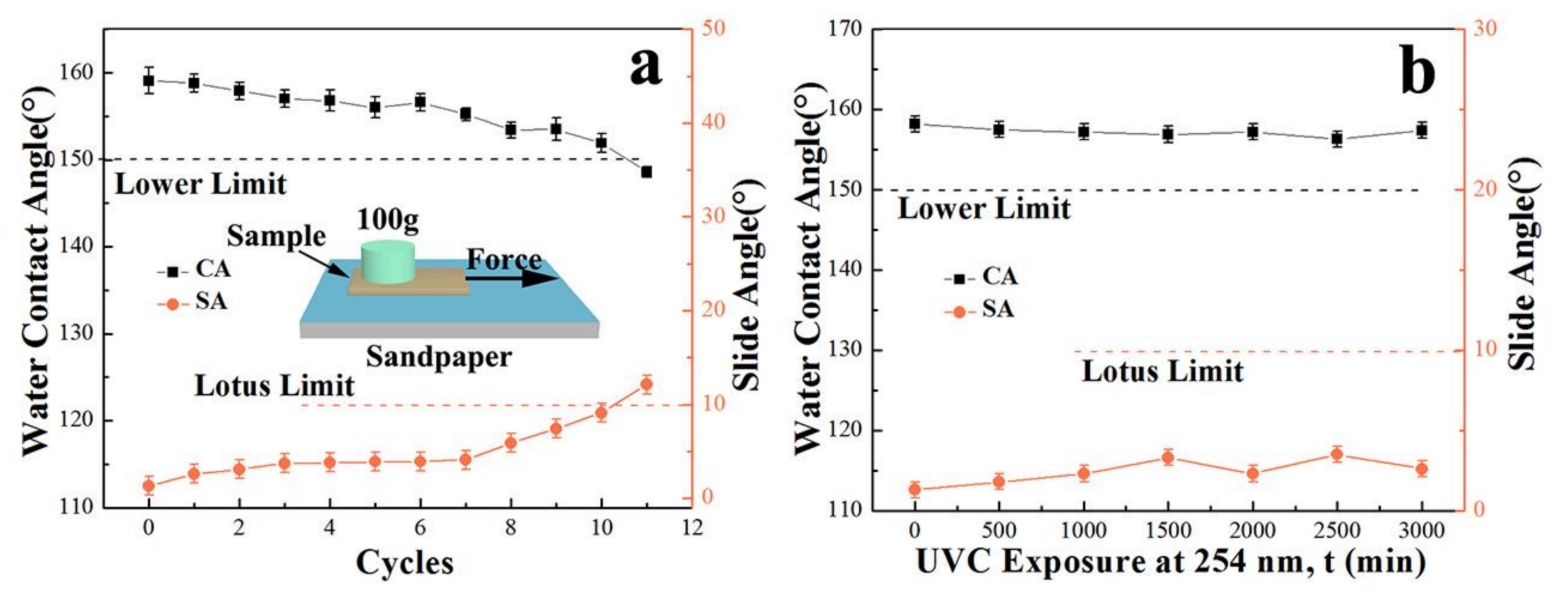
(**a**) The wear-resistant test process device (inset), and water CA as a function of the number of abrasion cycles for superhydrophobic coatings; (**b**) CA and SA changes of superhydrophobic coatings with ultraviolet (UV) irradiation time.

The mechanical durability of the conductive superhydrophobic coating is evaluated by the wear resistant test. The wear resistance testing method is shown in the inset of Figure 6a. The results show that within 10 cycles, the coating maintains its superhydrophobic property, showing excellent mechanical durability. This is because the micro-nanometer hierarchical structure is covered and reinforced by polyurethane resin, and the areas between microparticles of the surface and between the MWCNTs of surface are filled firmly with the polyurethane resin. However, after 10 cycles of friction, the micro-nanometer hierarchical structure is damaged and the superhydrophobic coating loses its superhydrophobic property accordingly. Figure 6b shows the CA and SA changes of the as-prepared superhydrophobic coatings with UV irradiation time. It was found that the contact angle and the sliding angle show no clear change after 50 h UV exposure, indicating the excellent resistance of the superhydrophobic coating to UV light.

## 4. Conclusions

It is reported for the first time that an environmentally friendly approach to fabricate conductive superhydrophobic coatings with sandwich-like structures of “SiWPU-MWCNTs-SiWPU” has been completed. This process is simple, safe, and avoids toxic solvent, fluoride material, and pollution. The functionalized multi-walled carbon nanotubes grafted with *N*-octadecylamine (MWCNTs-ODA) gain good dispersibility and stability in ethanol and create both microscale and nanoscale roughness, while the waterborne polyurethane modified AEAPS acts as a low surface energy material and a binder. The coating’s feature contact angle reaches 158.1° ± 2°, and the sliding angle is below 1°. Meanwhile, the coating shows good conductivity, electric heating characteristics, wear resistance, and UV resistance. Therefore, the multifunctional coating will have the potential for a wide application in the cold, outdoor environment and in devices that heat with electricity.

## Supplementary Materials

The following are available online at http://www.mdpi.com/2073-4360/10/4/378/s1, Video S1: The luminous effect of the conductive superhydrophobic coatings.
